# A Readmission Risk Assessment Tool Is Not Predictive of 90-Day Readmission After Total Joint Arthroplasty at an Urban Tertiary Referral Hospital

**DOI:** 10.7759/cureus.72651

**Published:** 2024-10-29

**Authors:** Akram Habibi, Ruijia Niu, Gloria S Coden, Hannah I Travers, Mikhail Kuznetsov, Geoffrey Stoker, Raminta Theriault, David Freccero, Eric L Smith

**Affiliations:** 1 Orthopedic Surgery, New York University (NYU) Langone Health, New York, USA; 2 Orthopedics, New England Baptist Hospital, Boston, USA; 3 Orthopedics, Beth Israel Deaconess Medical Center, Harvard Medical School, Boston, USA; 4 Orthopedics, Tufts Medical Center, Boston, USA; 5 Orthopedics, Boston Medical Center, Boston, USA

**Keywords:** emergency department readmission, healthcare financial burden, postoperative management, readmission risk assessment tool, total joint arthroplasty

## Abstract

Introduction: Readmission within 90 days of total joint arthroplasty (TJA) via an emergency department (ED) encounter represents a significant economic burden to the healthcare system. We aimed to determine the utility of a previously described readmission risk assessment tool (RRAT) in predicting readmission after presentation to the ED within 90 days of primary TJA.

Methods: At a single academic tertiary referral medical center, a retrospective chart review was used to collect demographic data, surgery type, medical history, reason for presentation in the ED, and ED disposition for the 1,576 patients who underwent TJA between April 1, 2016, and December 31, 2018. The RRAT score of patients was calculated and compared between patients who were discharged home versus readmitted to inpatient care.

Results: We identified 244 patients (328 encounters) who presented to the ED within 90 days of primary TJA, resulting in a 3.1% readmission rate. No statistical difference was found between the RRAT scores of readmitted and discharged patients (p=0.24). The most common reason for presentation to the ED for discharged patients was surgical site pain compared to medical concerns (cardiac, hematological, and renal concerns) in the readmitted group.

Conclusions: Although the RRAT score alone is not predictive of readmission within 90 days of TJA, the reason for presentation to the ED between discharged and readmitted patients does differ. These results present an opportunity for orthopedic surgery providers to discuss with other providers ways to optimize postoperative pain management and decrease readmissions. This study underscores the need for improved postoperative pain and chronic condition management to reduce ED visits and readmissions and highlights the necessity for larger, multi-center studies to better assess the RRAT score's predictive value.

## Introduction

Total hip and knee arthroplasty have been effective treatments for advanced arthritic disease by providing pain relief and improved quality of life for patients [1-5]. The number of total joint arthroplasty (TJA) surgeries performed has continued to increase, with some estimates showing more than a doubling of procedures performed over a 10-year period [6-7]. With an aging population in the United States, the number of TJAs is expected to continue increasing, with some studies predicting a 673% increase in total knee arthroplasties (TKA) and a 174% increase in total hip arthroplasties (THA) from 2005 to 2030 [8-9].

The rising total number of TJA surgeries performed will likely result in increased postoperative emergency department (ED) visits and readmissions. The current rates of readmissions after TJA have been reported to be between 4% and 8.5% in various studies [10-12]. The financial consequences of these ED visits and readmissions are of particular concern to healthcare institutions due to the current bundled payment system initiated by the Comprehensive Care for Joint Replacement model. In this system, hospitals are responsible for all of a patient’s care, including any related care within 90 days of the surgical encounter [13]. Previous studies have examined ED visits and readmissions after TJA [14-15]; they have identified that swelling and pain were common reasons for ED visits, while infection and medical complications often lead to readmission. The ability to predict which patients are at increased risk for readmission is an important way to limit the number of readmissions postoperatively and therefore decrease the financial burden to all stakeholders.

The readmission risk assessment tool (RRAT) is one example of a method to calculate the risk of readmission based on certain patient factors. Boraiah et al. sought to examine modifiable risk factors as predictive measures of readmission after hip and knee TJA [16]. The group assessed patients based on risk factors such as *Staphylococcus aureus* colonization, smoking, body mass index, cardiovascular disease, and diabetes history. Each risk factor was given a numerical scale. They risk-stratified patients undergoing TJA with their RRAT scoring system using a nested case-control design. Their study showed that an RRAT score greater than or equal to 3 was significantly associated with an increased risk of readmission. The purpose of the present study is to determine the usefulness of the RRAT score in predicting readmission after presentation to the ED within 90 days of primary TJA and to determine the reasons for presentation to the ED and readmission to the hospital at our institution.

## Materials and methods

After approval from the Institutional Review Board of Boston University Medical Center (approval number: H-38619), we conducted a retrospective cohort study (Level of Evidence III) and identified patients who underwent hip or knee TJA from April 1, 2016, to December 31, 2018, and presented to the ED within 90 days of surgery at a single academic tertiary rescue hospital. Patients were included if they underwent a primary TJA and presented to the ED within 90 days of surgery. For a majority of the patient group, surgery was performed for a primary diagnosis of osteoarthritis. Patients were excluded if TJA was performed for diagnosis of malignancy, if revision TJA was performed, or if presentation to the ED was more than 90 days after surgery. This left a total of 328 encounters (244 patients) included in the analysis.

The institutional electronic medical record was reviewed to collect the following data: age, sex, race, insurance status, procedure type, date of index procedure, ED encounter date, chief complaint for ED visit, ED disposition, reason for hospital readmission, and current relevant medical history including American Society of Anesthesiologists (ASA) score.

These patients were then divided into two groups based on whether the ED visit resulted in a hospital readmission or discharge. The demographic information was compared between the two groups using a t-test or Pearson Chi-square test. The RRAT score was calculated for the discharged and readmitted groups using the preoperative data outlined in Table 1 based on the methodology described in the previously referenced literature [16]. A two-tailed t-test was performed to compare the scores of both patient groups (discharged and readmitted patients). An alpha level of 0.05 was used. All statistical analysis was performed using R version 3.1.0 (R Foundation for Statistical Computing, Vienna, Austria, https://www.R-project.org/).

**Table 1:** Summary of risk factors and characteristics for the RRAT scoring BMI: body mass index, CAD: coronary artery disease, CVA: cerebrovascular accident, PVD: peripheral vascular disease, VTED: venous thromboembolic disease, COPD: chronic obstructive pulmonary disease, DVT: deep vein thrombosis, RRAT: readmission risk assessment tool

Risk factors	Characteristics	Score
*Staphylococcus aureus* status	Positive *Staphylococcus aureus* test	3
Smoking status	Current tobacco user	1
BMI (kg/m^2^)	30.0-34.9	1
	35.0-39.9	2
	>40.0	3
Cardiovascular disease	Patient >60 years old with a history of CAD, CVA, PVD, or VTED and two other risk factors: COPD, renal disease, diabetes, heart failure, hypertension, current smoker, and cancer	1
Venous thromboembolic disease	Pulmonary embolism or DVT	2
	Risk factors: CVA, COPD, BMI >40 kg/m^2^, CAD, PVD, activated protein C disease	1
Neurocognitive, psychological, or behavioral issues	Alcohol and/or narcotic abuse	2
	Neurocognitive deficiency	1
	History of depression	1
Physical condition	Non-ambulatory, i.e., wheelchair	2
	Comorbidities affecting ambulation	1
Diabetes	Preoperative glucose >180 mg/dl	3
	Hemoglobin A1C >8%	2
	Well-controlled diabetes	1

The reason for ED visits was further subdivided into the following categories based on previous studies in the literature [14-15]: cardiac, gastrointestinal, hematological, neurological, pulmonary, renal, complaints unrelated to surgery, wound concern, cellulitis, infection, complication of surgery, surgical site pain, fall, periprosthetic dislocation, and periprosthetic fracture, substance use/abuse, or other.

## Results

From April 1, 2016, to December 31, 2018, a total of 1,576 patients had a primary TJA at our institution and met the study inclusion criteria. Of these patients, 244 patients (328 encounters), accounting for 15.5% of the total cohort, had an ED encounter at our institution within 90 days of surgery. Of the encounters captured in the retrospective chart review, 53 encounters (49 patients, 3.1%) resulted in readmission to the hospital, and the remaining 275 ED encounters resulted in discharge home.

We compared the demographic information and preoperative comorbidities for those patients who were readmitted or discharged by the ED (Table 2). Patients who got readmitted by ED were significantly older than those who got discharged home (p<0.001). There was no statistically significant difference in all the other demographic characteristics between the two groups. We also evaluated and compared the RRAT scores. Although the overall RRAT score distribution did not differ between the two groups (p=0.24), the readmitted patients had higher scores on the subcategories for cardiovascular disease, venous thromboembolism (VTE), and neurocognitive conditions (p<0.001).

**Table 2:** Comparison of demographic and preoperative information for patients in ED readmission and ED discharge groups (N=224) ED: emergency department, SD: standard deviation, BMI: body mass index, ASA: American Society of Anesthesiologists, TKA: total knee arthroplasty, THA: total hip arthroplasty, RRAT: readmission risk assessment tool, MRSA: methicillin-resistant *Staphylococcus aureus, *VTE: venous thromboembolism, N/A: not applicable

Characteristics	ED readmission patients	ED discharged patients	p-value
n	36	186	N/A
Age (years) (mean±SD)	65.36±8.02	59.52±9.13	<0.001
Sex (male %)	30.60%	40.90%	0.33
BMI (kg/m^2^, mean±SD)	32.26±6.91	32.28±5.86	0.99
Race (%)			
Asian	2.80%	1%	0.72
African American	58.30%	47.30%	
Hispanic or Latino	2.80%	1.60%	
White	27.80%	24.20%	
Surgical site (%)			
TKA	24 (66.7%)	136 (73.1%)	0.56
THA	12 (33.3%)	50 (26.9%)	
ASA score (%)			
1	0 (0%)	0 (0%)	0.85
2	14 (38.9%)	68 (36.6%)	
3	21 (58.3%)	115 (61.8%)	
4	1 (2.8%)	3 (1.6%)	
Insurance status % distribution			
Commercial	26.50%	23.80%	0.46
Medicaid	55.90%	45.30%	
Medicare	17.60%	30.40%	
Others	0%	0.60%	
Diagnosis for surgery (%)			
Osteoarthritis	91.70%	89.80%	0.97
Length of stay (days, mean±SD)	4.22±4.17	3.49±2.59	0.17
Days since surgery (days, mean±SD)	28.23±25.59	29.89±24.60	0.71
RRAT score (% Patients)			
0	2.80%	2.70%	0.24
1	5.60%	15.10%	
2	8.30%	16.10%	
3	13.90%	17.70%	
4	22.20%	17.70%	
5	13.90%	14.00%	
6	13.90%	7.00%	
7	11.10%	2.20%	
8	5.60%	3.80%	
9	2.80%	3.20%	
10	0	0.50%	
RRAT score subcategories (mean±SD)			
MRSA score	0.50±1.13	0.61±1.21	0.61
Smoking score	0.28±0.45	0.32±0.47	0.64
BMI score	1.14±1.05	0.99±1.01	0.42
Ambulatory status score	0.64±0.54	0.76±0.51	0.18
Diabetes score	0.50±0.70	0.45±0.75	0.72
Cardiovascular score	0.33±0.48	0.06±0.25	<0.001
VTE score	0.83±0.81	0.28±0.58	<0.001
Neurocognitive score	0.78±1.02	0.15±0.49	<0.001

Table 3 depicts the reasons cited for patient ED visits. In the readmitted patient group, the most common reasons for ED encounters were more diverse and included cardiac (17.0%), hematological (11.3%), renal (11.3%), and pulmonary (9.4%) reasons. While in the discharged patient group, the two main reasons for ED encounters were surgical site pain (36.4%) and complaints unrelated to surgery (18.5%). More specifically, examples of specific clinical concerns addressed in the readmission group included diagnoses such as atrial fibrillation (cardiac reason), anemia (hematological), and deep vein thrombosis (hematological). Further analysis of the data demonstrated that the readmitted patient group had an overall increased incidence of infectious reasons for ED visits, including cellulitis (9.4%), fever (5.7%), infection (7.5%), and sepsis (3.8%). When combining both groups for analysis, surgical site pain (30.5%) was the most common reason for ED encounters postoperatively within 90 days of surgery.

**Table 3:** Comparison of reasons for ED encounters within 90 days after TJA between discharged and readmitted patients ED: emergency department, TJA: total joint arthroplasty

Reasons for visit	Readmitted (%) n=53	Discharged (%) n=275	Total reasons (%) n=328
Cardiac	9 (17.0)	6 (2.2)	15 (4.6)
Cellulitis	5 (9.4)	3 (1.1)	8 (2.4)
Complaints unrelated to surgery	3 (5.7)	51 (18.5)	54 (16.5)
Complication of surgery	1 (1.9)	14 (5.1)	15 (4.6)
Fall	0	13 (4.7)	13 (4.0)
Fever	3 (5.7)	6 (2.2)	9 (2.7)
Gastrointestinal	5 (9.4)	12 (4.4)	17 (5.2)
Hematological	6 (11.3)	3 (1.1)	9 (2.7)
Infection	4 (7.5)	5 (1.8)	9 (2.7)
Neurological	1 (1.9)	4 (1.5)	5 (1.5)
Other	1 (1.9)	9 (3.3)	10 (3.0)
Periprosthetic dislocation	0	1 (0.4)	1 (0.3)
Periprosthetic fracture	1 (1.9)	0	1 (0.3)
Pulmonary	5 (9.4)	8 (2.9)	13 (4.0)
Renal	6 (11.3)	5 (1.8)	11 (3.4)
Sepsis	2 (3.8)	0	2 (0.6)
Substance use/abuse	1 (1.9)	5 (1.8)	6 (1.8)
Surgical site pain	0	100 (36.4)	100 (30.5)
Swelling	0	16 (5.8)	16 (4.9)
Wound check	0	14 (5.1)	14 (4.3)

## Discussion

The financial burden of unexpected hospital visits can put a severe strain on the healthcare system, with the average cost of readmission for TJA patients ranging from $27,979 to $36,308 for TKA and THA, respectively [17]. In light of current bundled payment models, it is imperative to limit financial penalties on healthcare institutions by avoiding unplanned ED encounters and hospital readmissions within 90 days of TJA. Our study attempted to identify the reasons patients presented to the ED and were readmitted and whether the RRAT could be useful in predicting the likelihood of readmission after TJA at our institution. This study demonstrates that surgical site pain is a significant reason for patient presentation to the ED within 90 days of surgery, in particular, in the discharged group (36.4%). Strategies such as preoperative teaching about expectations as well as phone consultations have been shown to reduce the number of unexpected hospital visits due to issues related to surgical pain and pain management [18-19]. Implementing such preoperative education strategies at our institution may therefore help decrease the number of ED visits secondary to concerns of surgical site pain and decrease the financial burden of these postoperative ED visits.

Medical issues, especially cardiac and hematological, accounted for the majority of readmissions. Other studies have also found that a majority of readmissions after primary TJA are due to similar medical complications [11]. These results emphasize the importance of preoperative optimization and also the need for a team-based approach to help limit the ramifications these chronic medical conditions pose postoperatively. This may be accomplished by instituting a policy of timely follow-up with primary care providers and improved access to medical providers after surgery. This could transition the management of these chronic medical conditions to the outpatient setting instead of the financially burdensome emergency room or inpatient setting. For example, one study demonstrated a nearly 50% decrease in readmission by implementing postoperative strategies such as outpatient venous thromboembolic workups (instead of in the ED), early primary care follow-up after discharge (for management of chronic medical conditions), and physician education about financial ramifications of readmission [20].

In our analysis of the two groups, the calculated RRAT scores did not significantly differ between the discharged and readmitted groups (p=0.24). The RRAT score is based on a combination of preoperative screenings and medical comorbidities. Our study demonstrated that the subcategory scores for cardiovascular disease, VTE, and neurocognitive conditions were significantly higher for readmitted patients (p<0.001). This result coincides with Table 3, which demonstrated that patients who were more likely to be readmitted often presented for reasons related to other medical issues. The lack of a significant difference in the total RRAT scores between the groups in our population may be secondary to the relatively high frequency of medical comorbidities in both groups of our patient cohort. The results of our data, therefore, may not be applicable to all patient populations but may demonstrate some limitations to the use of the RRAT at tertiary rescue hospitals. This is especially relevant considering the weighted importance of medical comorbidities in the calculation of RRAT scores. In contrast, a study at an academic tertiary medical center demonstrated that underlying comorbidities did not lead to increased readmission rates, adding to the uncertainty of whether the primarily comorbidity-based RRAT score is truly of value in predicting readmissions in this patient population [21]. Other studies have demonstrated that readmissions postoperatively may be more related to inconsistencies in ED provider practice variability [22-23].

This study has several limitations. Our study was limited by its small sample size. There were only 53 encounters by 49 patients in our data set that resulted in readmissions through the ED within 90 days of TJA. Of the 53 encounters, only 36 had the necessary data to calculate an accurate RRAT score. If the sample size were larger, it is possible that the overall difference in the RRAT scores between the non-readmission group and the readmission group may become statistically significant. A total of 79 patients per group would be needed to achieve adequate study power. A larger sample size potentially would be more diverse than our single institution cohort and therefore further increase the validity of any results obtained. Performing the study at a single institution may also be a limitation in its own right. The population at our institution is unique and represents a medically underserved patient population. This fact could partly explain the increased frequency of medical reasons for readmissions, as our patients may have less consistent access to primary care providers than at other institutions, although medical reasons as a primary reason for readmission after TJA are consistent with other studies in the literature [11]. The uniqueness of our patient population may also limit the applicability of our results to other institutions and practices around the country.

Another limitation of this study was our data collection. Our data was collected retrospectively and based primarily on chart review, which may be affected by inaccurate or limited documentation. One specific limitation of the data we used was the lack of Patient Health Questionnaire (PHQ) scores recorded when calculating the RRAT score. The original applications of the RRAT described in the literature used a PHQ-9 score of greater than 7 to qualify for a diagnosis of depression16. For the calculation of the RRAT score in this study, a documented diagnosis of depression in the patient chart was used instead (Table 1). The difference in the definition of depression in the calculation of the RRAT score in this study may have affected the final calculated scores and therefore the validity of the results of the absolute values of the RRAT scores for the discharged and readmitted groups. It is less likely to affect the difference between the scores as the same technique was used for both the discharged and readmitted groups.

## Conclusions

The calculated RRAT score for patients presenting to the ED within 90 days after primary TJA did not significantly differ between the discharged and readmitted groups, although the overall score was slightly higher in the readmitted group. The main reason for presentation to the ED in the discharged group was surgical site pain, and the main reason for the readmitted group was for medical reasons. Although there were limitations with our study, it does highlight some important areas for improvement in healthcare. In particular, strategies focused on the management of postoperative pain, including preoperative education to improve expectations, may limit the financial burden of presentation to the ED. In addition, strategies to improve postoperative management of chronic medical conditions through improved patient access and utilization of outpatient medical providers for chronic medical conditions may prevent some portion of readmissions for medical concerns by transitioning the treatment of these conditions from the expensive inpatient setting to the outpatient setting when appropriate. Furthermore, larger, multi-center studies are needed to determine the true usefulness of the RRAT score in predicting readmission rates within 90 days of TJA.
